# Identification of Erythromycin and Clarithromycin Metabolites Formed in Chicken Liver Microsomes Using Liquid Chromatography–High-Resolution Mass Spectrometry

**DOI:** 10.3390/foods10071504

**Published:** 2021-06-29

**Authors:** Bo Wang, Soyeon Nam, Eunyeong Kim, Hayoung Jeon, Kiho Lee, Kaizhou Xie

**Affiliations:** 1 College of Veterinary Medicine, Yangzhou University, Yangzhou 225009, China; dz120180009@yzu.edu.cn; 2 College of Pharmacy, Korea University, Sejong 30019, Korea; nsy7809@gmail.com (S.N.); eunyeongkim@korea.ac.kr (E.K.); 2020020911@korea.ac.kr (H.J.); 3 Joint International Research Laboratory of Agriculture & Agri-Product Safety, Yangzhou University, Yangzhou 225009, China; 4 College of Animal Science and Technology, Yangzhou University, Yangzhou 225009, China

**Keywords:** nontargeted screening, LC/ESI-HR-MS, erythromycin, clarithromycin, metabolites, chicken liver microsomes

## Abstract

Nontargeted analysis can be used for the rapid screening and confirmatory analysis of veterinary drugs and their metabolites, which are important for the comprehensive safety evaluation of animal-derived foods. Here, a novel nontargeted screening approach based on liquid chromatography coupled with electrospray ionization–high-resolution mass spectrometry (LC/ESI–HR-MS) was developed to determine erythromycin, clarithromycin, and their metabolites in chicken liver microsomes. Erythromycin and clarithromycin were incubated in vitro in the presence of NADPH for 60 min to generate metabolites in chicken liver microsomes. After the incubation, the supernatant was extracted using ultrasonic shaking, orbital shaking, and centrifugation before analysis using LC/ESI-HR-MS in positive ion mode on an Agilent Eclipse Plus C18 column (100 mm × 2.1 mm; i.d. 3.5 µm) with 0.1 percent formic acid-water and acetonitrile as the mobile phases for gradient elution at 0.4 mL/min. The results show that erythromycin can produce N-desmethyl-erythromycin A in chicken liver microsomes, but clarithromycin cannot produce N-desmethyl-clarithromycin in chicken liver microsomes. The N-desmethyl-erythromycin A and N-desmethyl-clarithromycin were tentatively identified in chicken liver microsomes using the established quick analytic method, which will provide a theoretical foundation for future research on pharmacokinetics and drug elimination in poultry.

## 1. Introduction

Erythromycin (ERY) and clarithromycin (CLA) are macrolide antibiotics (MACs) with broad-spectrum antibacterial activity and have effects on both Gram-positive and Gram-negative bacteria. The antibacterial mechanism of ERY and CLA involves irreversible binding to the 50S subunit of the bacterial ribosome and selective inhibition of protein synthesis by blocking transpeptidation and mRNA displacement, which is the same as the antibacterial mechanism of chloramphenicol [1,2]. MACs are mainly used to treat diseases caused by aerobic Gram-positive and Gram-negative cocci, anaerobic bacteria, *Legionella*, *Mycoplasma*, and *Chlamydia*. Soluble erythromycin thiocyanate powder has been used as a veterinary medicine to treat chickens artificially infected with chronic respiratory diseases [3]. Although the toxicity of this type of antibiotic is low, unreasonable or excessive use can lead to contamination of animal-derived food and an increase in resistant strains that pose risks to humans. MACs and their metabolites will damage human health once swallowed and accumulated to a sufficient concentration in the body [4,5,6]. China, the United States, the European Union (EU), and the Joint FAO/WHO Expert Committee on Food Additives (JECFA) have defined maximum residue limits of 40–200 μg/kg ERY in milk, eggs, and edible tissues for the safety of animal-derived food and human health [7,8,9,10].

**Figure 1:**
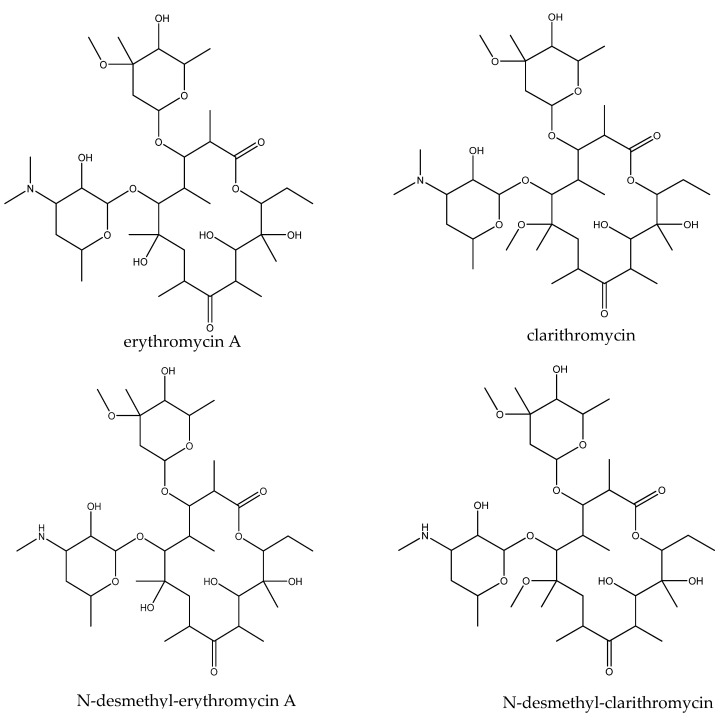
Chemical structures of erythromycin A, clarithromycin, N-desmethyl-erythromycin A, and N-desmethyl-clarithromycin.

The monitoring of veterinary drug metabolites in animal-derived foods has not received enough attention. However, studying such metabolite residues is extremely important for the safety of animal-derived foods. Phase I and phase II reactions are the two stages of drug metabolism in the liver. The first oxidation and reduction reactions in phase I are catalysed by monooxidases (mixed-function oxidases) in the liver. This group of enzymes constitute a complex microsomal system with haemoglobin cytochrome P-450 (CYP) as the core enzyme [11]. Studies have confirmed that ERY and other MACs undergo metabolic reactions under the action of cytochrome P-450 enzymes in liver microsomes in mice and humans and that ERY can undergo demethylation under the action of human and mouse liver microsomal CYP450 3A4 (CYP3A4) enzymes [11,12,13]. The CYP3A enzyme converts ERY into N-desmethylerythromycin and formaldehyde; therefore, the activity of CYP3A is proportional to the amount of formaldehyde formed. Nduka et al. [14,15] used this principle to study the effects of *Aframomum melegueta*, *Denniettia*, and *Millettia aboensis* extracts on CYP3A activity in intestinal and liver microsomes. The cytochrome P450 enzymes in liver microsomes of poultry (turkey, duck, quail, and chicken) that are orthologous to cytochrome P450 enzymes in human and mouse liver microsomes are CYP1A1/2, CYP2A6, and CYP3A4 [16,17,18,19]. Zhang et al. [20] reported the liver toxicity of macrolides with different structures in zebrafish and revealed their common targets for exerting hepatotoxic effects; this work will contribute to a better understanding of how macrolides should be used in clinical practice. Moreover, ERY and CLA have similar chemical structures (Figure 1), and both contain -N-(CH_3_)_2_ branches. In the present work, chicken liver microsomes containing CYP3A4 orthologues, the liver toxicity of MACs, and medicinal chemical structural factors were considered and ERY and CLA in chicken liver microsomes were incubated with N-demethylation metabolites.

The separation and detection characteristics of liquid chromatography tandem mass spectrometry (LC-MS/MS) make it ideal for the investigation of veterinary medication residues in complicated food matrices [21]. Many targeted analytical methods with high selectivity have been developed based on LC-MS or LC-MS/MS to detect MACs in fish, chicken, swine, bovine tissues, meat, eggs, and milk [6,22,23,24,25,26]. However, targeted methods can detect known compounds but cannot be used to identify unknown compounds [27,28]. To solve this problem, nontargeted screening based on LC-high-resolution (HR)-MS methods needs to be established for comprehensive analysis of known and unknown compounds [29,30]. HR-MS, as with quadrupole/time-of-flight (Q/TOF) or Q/orbital ion trap instruments, has the advantages of a wide scanning range, fast speed, and precise molecular weight determination, which enable retrospective data mining of a chromatogram to search for additional compounds of possible interest, such as metabolites [31,32]. Because of the efficient separation and high mass resolution, nontargeted screening based on LC-HR-MS methods will be useful for detecting veterinary drugs and their metabolites in animal-derived foods [33]. Kaufmann et al. [34] established a method for determining 100 veterinary medicines in various meat matrices using ultra high-performance liquid chromatography (UHPLC) coupled to TOF-MS. The UHPLC-TOF-MS method can detect 100 veterinary medicines simultaneously with good recovery and precision. Fu et al. [35] reported an LC-HRMS method to quickly screen and determine compounds that potentially pose health risks in meat samples and built an in-house risk substance (IHRS) database by summarizing the structural characteristics of specific classes of compounds. The IHRS database, which was established to quickly screen and detect unknown compounds and metabolites in animal-derived foods, contains approximately 500 different additives and drugs and was used to quickly screen unknown and suspicious chemicals in specific structure classes. Jia et al. [36] developed a nontargeted screening based on a UHPLC-HR (Orbitrap)-MS method to detect macrolides and metabolites in bass. HR-MS can accurately obtain the mass spectral ratio of a precursor ion and product ion and accurately determine the composition of the product ion fragment. Due to the high resolution and high sensitivity of this HR-MS method, an LC-HR-MS method was used to detect ERY, CLA, and their metabolites in chicken liver microsomes in this study. 

The purpose of this study was to verify whether ERY and CLA can produce N-desmethyl-erythromycin A and N-desmethyl-clarithromycin in chicken liver microsomes. Buspirone (BUS) can be metabolized by the CYP3A4 enzyme and thus was added as a positive control in this experiment. An in vitro incubation test was carried out in chicken liver microsomes, and a cofactor group and no cofactor (control) group were set up to verify the N-desmethyl-erythromycin A and N-desmethyl-clarithromycin. A nontargeted screening method based on LC-TOF-MS/MS was established to determine BUS, ERY, CLA, and their metabolites in chicken liver microsomes. This study will provide a scientific basis for the pharmacokinetics and drug elimination of ERY, CLA, and their metabolites in poultry and for the monitoring of MACs residues in animal-derived foods.

## 2. Materials and Methods

### 2.1. Chemicals and Reagents

Erythromycin A dihydrate (analytical standard), CLA (≥95% standard), buspirone hydrochloride, dimethylsulfoxide (DMSO, 1.100 g/mL), and the reduced form of β-nicotinamide dinucleotide phosphate (NADPH, ≥93% standard) were purchased from Sigma-Aldrich (St. Louis, MO, USA). N-Desmethyl-erythromycin A and N-desmethyl clarithromycin were purchased from Santa Cruz Biotechnology (Santa Cruz, CA, USA). Potassium phosphate buffer (0.1 M, pH 7.4) was purchased from Biosesang (Seongnam, Korea). HPLC-grade methanol and acetonitrile (ACN) were supplied by J.T. Baker Chemical (Radnor, PA, USA). Chicken liver microsomes (PL-MIC-201) were purchased from PRIMACYT GmbH (Schwerin, FRG, Germany) and stored at −80 °C before use. All chemicals were of the highest purity available.

### 2.2. Preparation of the Standard Stock and Working Solutions

Standard 20 mM stock solutions of erythromycin A dihydrate, buspirone hydrochloride, N-desmethyl-erythromycin A, and N-desmethyl-clarithromycin were prepared in pure methanol. A standard 20 mM stock solution of CLA was prepared in DMSO. These standard stock solutions were stored at −20 °C. Standard working solutions of erythromycin A dihydrate, CLA, buspirone hydrochloride, N-desmethyl-erythromycin A, and N-desmethyl-clarithromycin at 20 μM were prepared by diluting the stock solutions with 0.1 M potassium phosphate buffer (pH 7.4). The standard working solutions were prepared for immediate use through gradual dilution. A 10 mM NADPH solution was prepared in 0.1 M potassium phosphate buffer (pH 7.4). An internal standard (IS) solution of 1 μg/mL CLA and an IS solution of 1 μg/mL erythromycin A dihydrate were prepared in pure ACN and stored at −20 °C.

### 2.3. Microsomal Incubations

The incubation system (100 μL) contained 65 μL of potassium phosphate buffer (0.1 M, pH 7.4), 20 μL of chicken liver microsomes (5 mg/mL), 5 μL of a compound (20 μM) and 10 μL of NADPH (10 mM). After a 5 min preincubation period at 37 °C, the reaction was started by introducing 10 μL of NADPH (10 mM) as the cofactor solution. The final study groups were divided into cofactor and no cofactor (control) groups. After preincubation for 5 min, 10 μL of potassium phosphate buffer (0.1 M, pH 7.4) was added to the control group. At 0 and 60 min, the reaction was quenched with 100 μL of ice-cold ACN with IS (1 μg/mL; CLA and ERY for ERY and CLA, respectively). For 0 min samples, the reaction was quenched immediately after the 5-min preincubation, followed by the addition of 10 μL of NADPH (10 mM) and 10 μL of potassium phosphate buffer (0.1 M, pH 7.4) to the cofactor and control groups, respectively. Following brief sonication (5 min) and vortexing (5 min), the mixtures were centrifuged for 30 min at 3000 rpm and 4 °C. The supernatant was transferred to an Agilent 96-well plate and then analysed on an Agilent 6530 Q-TOF LC-MS/MS instrument.

### 2.4. LC/ESI-HR-MS Analysis

Liquid chromatographic separation and mass spectrometric detection were performed on an Agilent 1200 series HPLC system and an Agilent 6530 Q-TOF LC-MS/MS system equipped with a dual AJS ESI ion source. Chromatographic separation was performed on an Agilent Eclipse Plus C18 column (100 mm × 2.1 mm; i.d. 3.5 µm) by gradient elution with a mobile phase consisting of 0.1 vol.% formic acid in water (A) and ACN (B) at 40 °C: 0–2 min, 5 vol.% B; 2–3 min, 50 vol.% B; 3–4 min, 75 vol.% B; and 4-5 min, 5 vol.% B. The injection volume was 5 μL with a flow rate of 0.4 mL/min. Before sample injection, the instrument was equilibrated under the initial gradient conditions (A:B = 95:5, *v/v*) and the above-mentioned column temperature and flow rate conditions. HR-MS detection was carried out in positive ionization mode with the following ion source parameters: capillary voltage, 4000 V; drying gas, 12 L/min and 325 °C; nebulizer gas, 35 psi; and sheath gas, 10 L/min and 350 °C. Mass spectra were acquired in positive ion auto MS/MS mode (F, 100 V; CE, 0 eV) with a full scan range of m/z (100–1000) and a mass scan rate of 5 spectra/s. The mass spectrometer was run in an extended dynamic range mode with a mass range of m/z 50–3200. The mass resolving power was greater than 12,000 FWHM at m/z 1552. 

### 2.5. Data Analysis

The data were analysed using Agilent MassHunter Quantitative Analysis software (version B.05.00) and Agilent MassHunter Metabolite ID software (version B.04.00) (Santa Clara, CA, USA). The remaining content was evaluated by dividing the ratio of the response of the target compound to the response of the IS at 60 min of incubation by the ratio of the response of the target compound to the response of the IS at 0 min of incubation in the cofactor group or control group.

## 3. Results and Discussion

**Figure 2:**
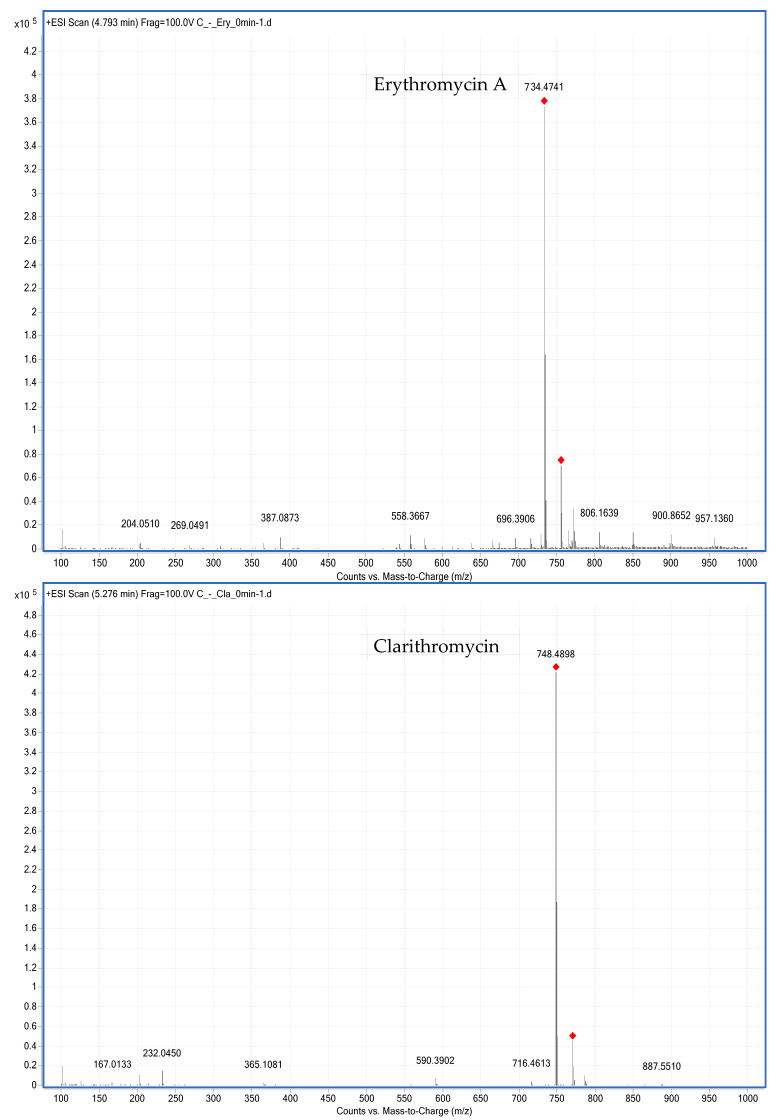
MS spectrums of erythromycin A and clarithromycin.

**Figure 3:**
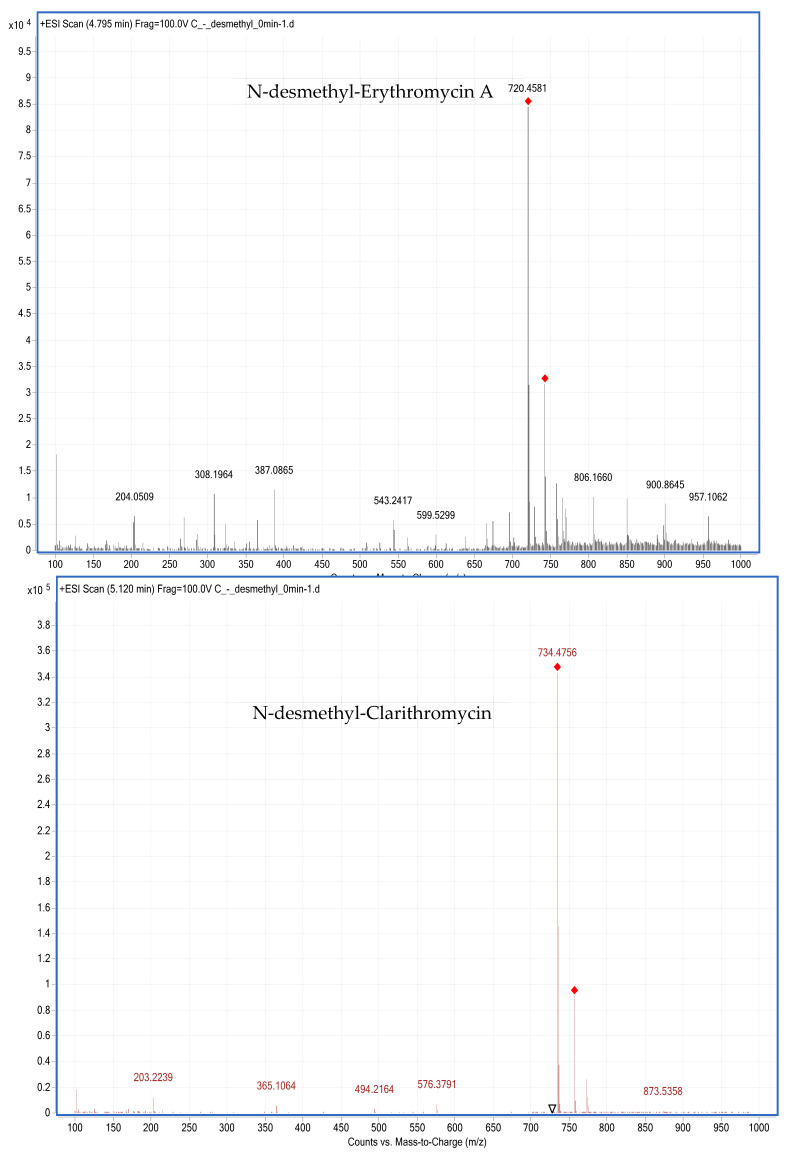
MS spectra of N-desmethyl-erythromycin A and N-desmethyl-clarithromycin.

In the existing literature, C18 columns are used predominantly in LC-MS/MS methods for determining MACs and their metabolites in animal-derived foods [6,22,24,25,26,36]. BUS, erythromycin A, CLA, and their metabolites are polar compounds, and a C18 column can retain polar compounds well and achieve good separation. Therefore, an Agilent Eclipse Plus C18 column (100 mm × 2.1 mm; i.d. 3.5 µm) was used to determine BUS, erythromycin A, CLA, and their metabolites in chicken liver microsomes. In this study, the mobile phase consisted of 0.1 vol.% formic acid in water-ACN, and the gradient elution conditions were optimized so that the target compound produced a good peak shape without tailing. In MS mode, the precursor ions for BUS, erythromycin A, CLA, N-desmethyl-erythromycin A, and N-desmethyl-clarithromycin are protonated molecular ions [M + H]^+^ at m/z 386.2577, 734.4741, 748.4898, 720.4581, and 734.4756 (Figure 2 and Figure 3), respectively, in positive ionization mode.

ERY and CLA are effective inhibitors of the cytochrome P450 system (especially CYP3A4). Midazolam was used as the CYP3A4 substrate to evaluate the strength of drug interactions mediated by CLA and ERY. CLA and ERY caused strong and moderate interactions, respectively, possibly because the strengths of their inhibition of the main drug-metabolizing enzyme CYP3A4 differ but they do not inhibit the activity of the drug transporter P-GP [37,38]. Chicken liver microsomes contain human isoenzymes of CYP1A2, CYP2C9, CYP2C19, CYP2D6, and CYP3A4, which were evaluated by PRIMACYT GmbH (Schwerin, FRG, Germany). The absence of clear orthology between avian and human CYP2C or CYP3A genes, as well as the occurrence of CYP2J, CYP2AB, and CYP2AC duplication events in the early bird lineage, were discovered by Watanabe et al. [17]. The contribution of each CYP subtype to drug metabolism largely depends on its protein expression level in the liver. ERY and CLA are commonly used to treat chronic respiratory diseases in chickens. Moreover, ERY is metabolized by enzymes of the cytochrome P450 system, especially isoenzymes of the CYP3A superfamily. Therefore, this study verified whether ERY and CLA produced N-desmethyl-erythromycin A and N-desmethyl-clarithromycin in chicken liver microsomes.

Based on previous studies by Zhou et al. [39] and Lee et al. [40], we designed an in vitro incubation test. BUS is an anti-anxiety medication that alters brain chemicals that may be out of balance in persons who suffer from anxiety. Tension, dizziness, pounding heartbeat, and other physical symptoms of anxiety are treated with BUS. The enzyme CYP3A4 has been demonstrated to metabolize BUS in vitro [41]. This conclusion is in line with the in vivo interactions found between BUS and CYP3A4 inhibitors or inducers [42]. Therefore, we added BUS to chicken liver microsomes for in vitro incubation experiments to study the related metabolism. After preincubation for 5 min, the cells were incubated for 0 min or 60 min after adding the IS, and then 10 μL of NADPH (10 mM) and 10 μL of potassium phosphate buffer (0.1 M, pH 7.4) were added to the cofactor and control groups, respectively. The purpose of this procedure was to decrease the drug metabolism of erythromycin A at 0 min of incubation to more accurately quantitate the remaining amount of erythromycin A after 60 min of incubation.

**Figure 4:**
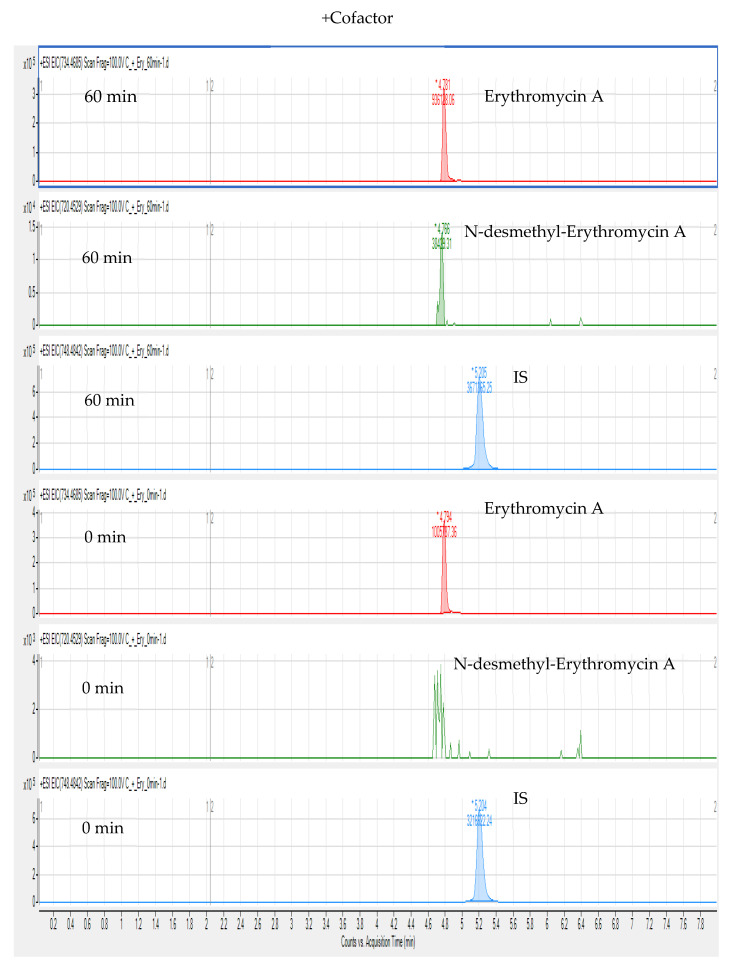
Extracted ion chromatograms of erythromycin A, N-desmethyl-erythromycin A, and IS after adding NADPH and incubating for 60 and 0 min.

**Figure 5:**
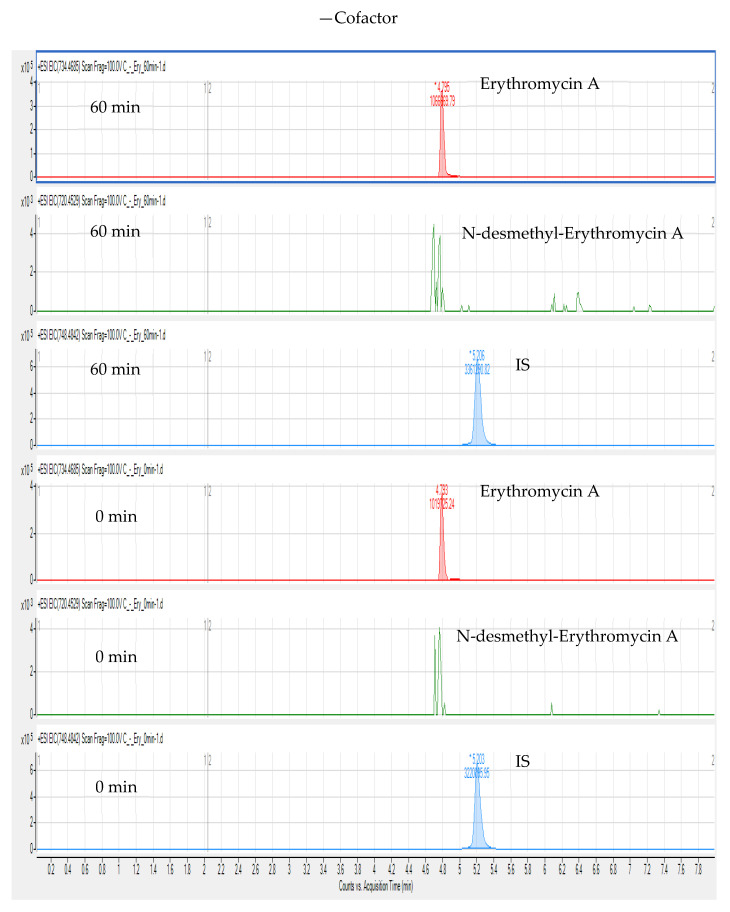
Extracted ion chromatograms of erythromycin A, N-desmethyl-erythromycin A, and IS without adding NADPH and incubating for 60 and 0 min.

Compared with targeted and quantitative approaches, a nontargeted screening method based on matching fragmentation characteristics can confirm and analyse unknown substances and collect as much substance information as possible, with wide coverage. Thus, this study used a nontargeted screening method based on LC-HR-MS to detect BUS, erythromycin A, CLA, and their metabolites in chicken liver microsomes. We extracted the ion chromatograms of BUS, CLA (IS), and N-desmethyl-erythromycin A in chicken liver microsomes at m/z 386.2551, 748.4842, and 720.4529 (calculated), respectively. In the extracted ion chromatograms, the retention times of BUS, IS, and N-desmethyl-erythromycin A are 4.70, 5.22, and 4.77 min, respectively. The extracted ion chromatograms of erythromycin A (calculated m/z 734.4685), N-desmethyl-erythromycin A, and IS from the cofactor and control groups at 60 min and 0 min incubation are shown in Figure 4 and Figure 5. In contrast to Figure 5, Figure 4 shows that erythromycin A produced N-desmethyl-erythromycin A in chicken liver microsomes after incubation for 60 min and addition of NADPH cofactor, but there was no peak corresponding to N-desmethyl-erythromycin A under other conditions.

**Figure 6:**
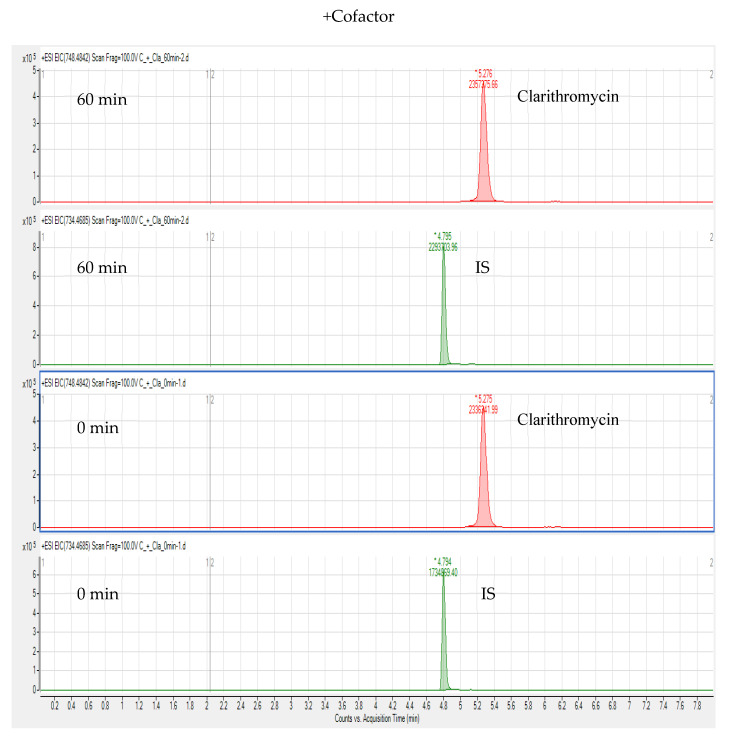
Extracted ion chromatograms of clarithromycin and IS after adding NADPH and incubating for 60 and 0 min.

**Figure 7:**
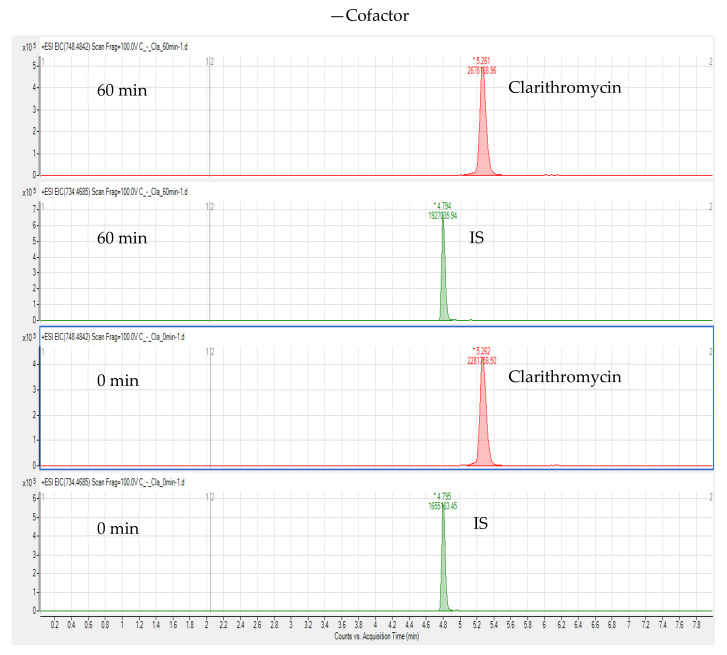
Extracted ion chromatograms of clarithromycin and IS without adding NADPH and incubating for 60 and 0 min.

The in vitro incubation test of CLA in chicken liver microsomes was carried out based on erythromycin A; erythromycin A was used as an IS and detected by LC-HR-MS. We extracted the ion chromatograms of BUS, IS, and N-desmethyl-clarithromycin (calculated m/z 734.4685) in chicken liver microsomes, and the retention times of these compounds were 4.71, 4.80, and 5.12 min, respectively. Figure 6 and Figure 7 show that N-desmethyl-clarithromycin was not extracted under any of the conditions. Therefore, CLA cannot produce N-desmethyl-clarithromycin in chicken liver microsomes, possibly because CLA is a strong inhibitor of CYP3A4, which inhibits the production of N-desmethyl-clarithromycin by the CYP3A4 isoenzyme in chicken liver microsomes.

Residues of erythromycin A, N-desmethyl-erythromycin A, and CLA in animal-derived foods can pose human health risks due to toxicity. Therefore, drug metabolism residues in animal-derived foods should be considered. This study evaluated the levels of erythromycin A and CLA metabolism remaining in chicken liver microsomes, as shown in Table 1. After incubation for 60 min and the addition of NADPH cofactors, the average residual contents of BUS, ERY, and CLA in chicken liver microsomes were 48.9%, 86.6%, and 89.8%, respectively. After 60 min of incubation but no addition of NADPH, BUS, ERY, and CLA were metabolized to certain degrees.

## 4. Conclusions

In this study, whether erythromycin A and CLA produce N-desmethyl-erythromycin A and N-desmethyl-clarithromycin in chicken liver microsomes was tentatively identified by LC-HR-MS. Through comparison of the extracted ion chromatograms with standard chromatogram, it was found that erythromycin A can produce N-desmethyl-erythromycin A in chicken liver microsomes, but CLA cannot produce N-desmethyl-clarithromycin. Nontargeted screening based on the LC-HR-MS method is fast, efficient, and sensitive and is suitable for the detection of veterinary drugs and their metabolites in animal-derived foods. This study will provide a scientific basis and detection methods for the monitoring of erythromycin A, CLA, and N-desmethyl-erythromycin A in poultry.

## Figures and Tables

**Table 1:** BUS, ERY, and CLA metabolize the remaining amount in chicken liver microsomes.

Analyte	Matrix	+Cofactor			Mean ± SD (%)	−Cofactor			Mean ± SD (%)
		Remaining Content (%)				Remaining Content (%)			
		1	2	3		1	2	3	
BUS	CLM	46.4	46.2	54.1	48.9 ± 4.5	87.5	65.7	91.2	81.5 ± 13.8
ERY	CLM	82.6	83.1	94.1	86.6 ± 6.5	99.0	97.9	87.6	94.8 ± 6.3
CLA	CLM	77.5	77.3	114.7	89.8 ± 21.5	100.7	78.7	80.3	86.6 ± 12.3

Abbreviations: buspirone, BUS; erythromycin A, ERY; clarithromycin, CLA; chicken liver microsomes, CLM.

## Data Availability

Data are contained within the article. The data presented in this study are available in the present article.
